# Thermodynamic Limitations and Exergy Analysis of Brackish Water Reverse Osmosis Desalination Process

**DOI:** 10.3390/membranes12010011

**Published:** 2021-12-23

**Authors:** Alanood A. Alsarayreh, Mudhar A. Al-Obaidi, Alejandro Ruiz-García, Raj Patel, Iqbal M. Mujtaba

**Affiliations:** 1 Department of Chemical Engineering, Faculty of Engineering, Mutah University, Al Karak 00962, Jordan; alanoodalsarayreh@gmail.com; 2 Department of Computer Techniques, Technical Institute of Baquba, Middle Technical University, Baquba 00964, Iraq; dr.mudhar.alaubedy@mtu.edu.iq; 3 Department of Electronic Engineering and Automation, University of Las Palmas de Gran Canaria, 35017 Las Palmas de Gran Canaria, Spain; 4 Department of Chemical Engineering, Faculty of Engineering and Informatics, University of Bradford, Bradford BD7 1DP, UK; R.Patel@bradford.ac.uk (R.P.); I.M.Mujtaba@bradford.ac.uk (I.M.M.)

**Keywords:** desalination, brackish water, reverse osmosis, exergy analysis, exergy distribution

## Abstract

The reverse osmosis (RO) process is one of the most popular membrane technologies for the generation of freshwater from seawater and brackish water resources. An industrial scale RO desalination consumes a considerable amount of energy due to the exergy destruction in several units of the process. To mitigate these limitations, several colleagues focused on delivering feasible options to resolve these issues. Most importantly, the intention was to specify the most units responsible for dissipating energy. However, in the literature, no research has been done on the analysis of exergy losses and thermodynamic limitations of the RO system of the Arab Potash Company (APC). Specifically, the RO system of the APC is designed as a medium-sized, multistage, multi pass spiral wound brackish water RO desalination plant with a capacity of 1200 m^3^/day. Therefore, this paper intends to fill this gap and critically investigate the distribution of exergy destruction by incorporating both physical and chemical exergies of several units and compartments of the RO system. To carry out this study, a sub-model of exergy analysis was collected from the open literature and embedded into the original RO model developed by the authors of this study. The simulation results explored the most sections that cause the highest energy destruction. Specifically, it is confirmed that the major exergy destruction happens in the product stream with 95.8% of the total exergy input. However, the lowest exergy destruction happens in the mixing location of permeate of the first pass of RO desalination system with 62.28% of the total exergy input.

## 1. Introduction

Due to the scarcity of freshwater resources, the improvement of water desalination technologies is by far the most important target for current researchers aiming to deliver potable water [1,2]. Furthermore, there is an exponential demand for water for domestic, agricultural, industrial, and other applications. The main desalination technologies are the reverse osmosis (RO) and multi-stage flash (MSF) processes, which account for almost 87% of the total fraction of freshwater produced in the world [3]. The other water desalination technologies of multiple effect desalination (MED), electrodialysis, and vapor compression share the remaining fraction of 10% of freshwater produced [4].

The RO process has dominated the membrane technologies due to it producing freshwater at a reduced energy consumption. Indeed, the simple design of compact size and modularity of RO units and ease of operation at an ambient temperature together with other merits have made the RO process the most popular desalination technology [5,6]. The flexibility in capacity expansion of the RO process with short construction periods, low investment costs and low periodical maintenance enables ease of construction of RO desalination plants of different sizes in the rural areas of water shortage. However, the performance capacity of the RO process is directly related to the inlet conditions and therefore it varies based on the feed water quality [7], site location and the start-up and shut-off [8,9].

The main principle of RO process is to treat poor quality (high salinity) water and produce high quality freshwater and disposed brine. This is originally occurring due to using higher pressure than the osmotic pressure that causes water passage through the membrane pores from the high concentration side into the low concentration side, complemented by the rejection of majority salts [10,11].

The RO process, and especially the large-scale RO systems, require a vast amount of energy to operate the high pressure pumps in all the modularized units, resulting in severe exergy destruction [12]. In this regard, the necessity of energy is directly related to the operating conditions where an increase in the feed salinity or pressure would cause an increase in the supplied energy [13,14]. Although the RO process requires less than half the energy needed for thermal processes [15], research on improving the existing water desalination plants is progressively increasing to introduce the RO process as the most reliable, efficient and economical option compared to other involved water treatment technologies [16,17]. In this regard, the brackish and seawater water RO desalination systems consume about 3.6 kj/kg, and 5.4 kj/kg to 9 kj/kg, respectively [18]. The main intention of the recent research studies was to investigate the most reliant parts of energy dissipation. The exergy based on thermodynamic properties was on top of other methodologies used to explore the energy losses in actual RO desalination plants [17]. In any process, exergy differs from energy in that exergy is always destroyed and not conserved. More importantly, the thermodynamic imperfections of any industrial process cannot be measured by energy calculations (first law of thermodynamic), unlike exergy which signifies the causes of energy losses and justifies the irreversibilities such as chemical reactions, heat transfer through a finite temperature difference, friction, mixing, and unrestrained via the second law of thermodynamics [19].

Exergy and energy analyses have gradually attracted greater attention to achieve the requirements for thermodynamic calculations with high accuracy [20]. This is due to the complexity and performance of power-generating units which significantly increased and improved to manage the depletion of fossil fuel resources and reduce the environmental impacts. Basically, the aim of an exergy analysis is to improve the thermodynamics of the process by offering an option to reduce the electrical energy consumption and therefore making the desalination plant more cost effective. In other words, investigating the main sources of exergy dissipation would conceivably reduce the total energy consumption [21,22].

Aljundi [19] stated that the exergy analysis is a potential tool for specifying the inadequacies of the RO process, which can aid to improve the overall performance. Exergy enables the appraisal of maximum work that can be extracted from a specific system relative to the surrounding environment [1]. The following section presents some examples of these articles.

Cerci [16] conducted an exergy analysis of an RO desalination plant in California, with capacity 7250 m^3^/d, by use of actual operational data of the plant. The exergy destruction distribution was evaluated, and the results indicated that the largest exergy destruction in the membrane modules occurred with about 74.07% of the total exergy input. Moreover, the mixing chamber got the smallest exergy destruction of 0.67% of the total exergy input.

Kahraman et al. [23] examined the exergy destruction rates and exergy flow rates in a brackish water RO desalination plant. They confirmed that the most exergy destruction is attributed to motors, pumps, and the separation units. Statistically, 39.7% of the total exergy is destructed in the pump/motor of the RO unit. However, the cost of desalination can be reduced drastically via the implementation of efficient pumps. Generally speaking, the second law efficiency of an RO system is about 8%.

Aljundi [19] evaluated thermodynamically the exergy flow rates and the exergy destruction rates for the RO plant of Al-Hussein thermal power station located in Jordan. The work showed that the exergy destruction occurred within the throttling valves, the pumps, and the motors with rates of 56.8%, 21%, and 19.6%, respectively. The second law efficiency was found to be quite low, at about 4.1%. Therefore, Aljundi [19] highlighted the importance of employing high-efficiency pump/motor combinations together with energy recovery devices besides the replacement of the existed throttling valves.

El-Emam and Dincer [1] examined the RO seawater desalination plant performance based on the first and second laws of thermodynamics. The results showed that the largest amount of irreversibility arises within the high-pressure pump (17.16%) and in the RO module (67.8%). However, the overall system exergy efficiency is about 5.82%, and the exergy destruction limited to 35.5% using an energy recovery device of a Pelton turbine as compared with using an expansion valve. More recently, Fellaou et al. [17] analysed the exergy destruction of a full-scale RO desalination system located in the Canary Island, Spain. This analysis revealed that the membrane modules have the largest thermodynamic losses of 64.28%, whilst he high-pressures pump and the feed pump accounted for losses of 40.84% and 38.48%, respectively.

The above research confirmed the possibility of mitigating the thermodynamic limitations of RO systems by identifying the units responsible for dissipating the energy. However, no work has been found in the literature that has analysed the exergy losses and thermodynamic limitations of the medium-sized industrial RO system of the Arab Potash Company (APC). Specifically, the RO system of the APC is characterised by two different configurations of retentate reprocessing design in the first pass and the permeate reprocessing design in the second pass to produce a high-quality water. Therefore, this study aims to resolve this challenge by carrying out a thorough exergy analysis based on chemical and physical exergies for all the main parts of the RO desalination plant. A successful model was obtained from the open literature to carry out the calculations of chemical and physical exergy and exergy flow rate. It is fair to expect that the methodology presented in this study would be a suitable option to determine the most important parts of the RO system of the APC that need to be considered for further improvement. It is also important to note that the same authors have developed a specific mathematical model for the RO system of the APC besides evaluating several methods to improve the performance indicators in a series of published articles. Thus, this study aims to appraise the thermodynamic limitations of RO system in an attempt to enhance the process.

## 2. Thermodynamic Limitations and Exergy Analysis of Brackish Water RO Desalination Plant of the APC

This section focuses on analyzing the thermodynamic limitations and exergy destruction of the multistage multi pass brackish water RO desalination system of the Arab Potash Company (APC). The examined parts of RO system include: (1) the RO membrane modules in which the saline water is separated into the permeate and retentate; (2) the throttling valve where the pressure of liquid is reduced; (3) mixing zones where the permeates or retentate streams are mixed; and (4) various process components such as pumps, disposal water stream, and product water stream. To carry out this study, a sub-model of exergy analysis was obtained from literature as presented in Section 2.2.

### 2.1. General Overview of the RO System of the APC and Tested Locations

The RO desalination system of the APC was constructed to desalinate the brackish water of feed salinity 1098.62 ppm and flow rate of 1776 m^3^/day. The pure water produced is demineralized by ion exchangers and directly fed to the boilers. The RO plant produces 1200 m^3^/d equivalent to 13.85 kg/s of pure water with salinity around (2 ppm).

**Figure 1:**
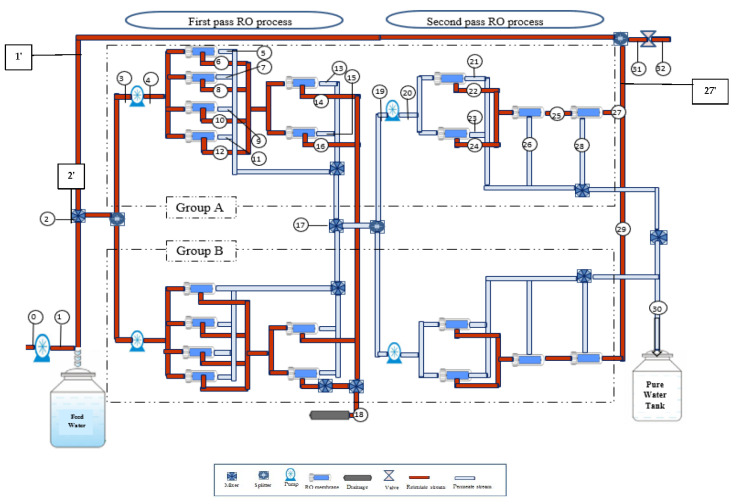
Flow diagram of the multistage RO brackish water desalination plant of the Arab Potash Company.

At this point, it is worth noting that there are two groups (A,B) in the first and second passes of RO process, which have the same number of RO membranes and are arranged in parallel as presented in the flow diagram of the plant in Figure 1. At position (2), the brackish raw water is fed to the plant with a flow rate of 1776.00 m^3^/day, which contains 1536 m^3^/day raw feed water (position 1) mixed with a recycled retentate of 240 m^3^/day (position 1′). The mass feed flow rate is divided into two parallel streams (groups A,B) called stages, and then pumped with high pressure pumps to the RO membrane modules in stages A and B. The membrane modules require high operating pressure to overcome the fluid friction and osmotic pressure that occurs across the membranes. The high-pressure pump is used to raise the pressure of the feed water from 923.071 kPa at position (3) to 934.22 kPa at position (4). The retentate water from the RO modules (6, 8, 10 and 12) (stage A) are companied and fed to the next stage in the first pass of RO process to produce a retentate at (14,16), and then is discharged into the drainage system at position (18) after being mixed with the collected retentate of stage B. However, the permeate water streams (5, 7, 9, and 11) and permeate from next parallel stages (13, 15) are mixed with the permeate of stage B at position (17). Then, this permeate is fed into the membrane modules in first stage of second pass RO process by two high pressure pumps. Next, the permeates from all RO modules of second pass (21, 23, 26, and 28) are mixed with the permeate of the stage B and collected in a product tank at position (30) with a daily production capacity of 1200 m^3^/day of around 2 ppm salinity. The retentate results from the first stage composed of two RO modules (22, 24) is fed to the second stage (one RO module) and then fed to the third final stage (25) of one RO module. Finally, the retentate from the third final stage of second pass (27) of group A is mixed with retentate of group B at position (29) and recycled back to be mixed with plant feed raw water at position 27′. The recycled flow passes through a throttling valve (31).

The Toray membrane USA is used here. The membrane characteristics are provided in Table 1. The pumps (type: Goulds pumps, ITT) are multistage vertical centrifugal with wetted parts constructed from 316 SS material and a motor efficiency of 91.7%.

### 2.2. Mathematical Model: Exergy Analysis

The RO brackish water desalination plant of the APC performs an efficient separation to produce fresh water from a resource of brackish water. The feed water is separated into retentate water (brine) and product water (low salinity water). Based on this, the analysis of salt and pure water properties must be considered. Salinity is typically conveyed in parts per million (ppm). The mole fraction of salt *x_s_* is determined from the following relations [24]:

(1) $$mf_{s}=\frac{m_{s}}{m_{\mathrm{mix}}}=\frac{N_{s}M_{s}}{N_{\mathrm{mix}}M_{\mathrm{mix}}}=x_{s}\frac{M_{s}}{M_{\mathrm{mix}}}$$

(2) $$mf_{w}=x_{w}=1-mf_{s}$$

(3) $$Mf_{w}=\frac{M_{w}}{M_{\mathrm{mix}}}$$

where *mf*, *M*, *N*, and *x* are the mass fraction, molar mass, number of moles, and the mole fraction, respectively. The subscripts s, w, and mix represent the salt, water, and mixture of saline water, respectively.

The molar mass of the saline water can be expressed as:

(4) $$M_{m}=\frac{m_{m}}{N_{m}}=\frac{N_{s}M_{s}+N_{w}M_{w}}{N_{m}}=x_{s}M_{s}+x_{w}M_{w}$$

Combining Equations (1)–(4) yields Equations (5) and (6) to convert the mass fractions into mole fractions. The molar masses of water and NaCl are 18.0 kg/kmol, and 58.5 kg/kmol, respectively.

(5) $$x_{w}=\frac{M_{s}}{M_{w}\left(\frac{1}{mf_{w}}-1\right)+M_{s}}=\frac{58.5}{18\left(\frac{1}{mf_{w}}-1\right)+58.5}=1-x_{s}$$

(6) $$x_{s}=\frac{M_{w}}{M_{s}\left(\frac{1}{mf_{s}}-1\right)+M_{w}}=\frac{18}{58.5\left(\frac{1}{mf_{w}}-1\right)+18}=1-x_{w}$$

The salinity of brackish water of the APC plant is 1098.62 ppm. Thus, the salt and water mass fractions are *mf*_s_ = 0.001098 and *mf*_w_ = 0.99890138, since ppm = *mf*_s_ × 10^6^, respectively. Additionally, the mole fractions are calculated from Equations (5) and (6), to be *x*_s_ = 3.383 × 10^4^ and *x*_w_ = 0.9997, respectively.

The average salinity of brackish water of the APC is 0.109862% and therefore it can be considered as diluted solution since the salinity is lower than 4%. The diluted solution can behave, to a large extent, as an ideal solution where it is reasonable to ignore the consequence of molecules of salt and water (dissimilar molecules) on each other.

The extensive properties per unit mass of a mixture can be represented by enthalpy *h* (kJ/kg) and entropy *s* (kJ/kg K). These properties can be resolute by the sum of each individual component in a mixture at a specified temperature and pressure [25] as depicted in the following expressions:

(7) $$h={\sum mf_{i}h}_{i}=mf_{s}h_{s}+mf_{w}h_{w}$$

(8) $$s={\sum mf_{i}s}_{i}=mf_{s}s_{s}+mf_{w}s_{w}$$

where *h*_s_, *s*_s_, *h*_w_ and *s*_w_ are the specific enthalpy of salt (kJ/kg), specific entropy of salt (kJ/kg K), specific enthalpy of water (kJ/kg), and specific entropy of water (kJ/kg K), respectively.

The inlet conditions of brackish water are presented in Table 2. These values will be taken as the properties at the dead state.

The enthalpy and entropy of salt and water at a given temperature T (K) can be specified from the following relations [26]:

(9) $$h_{s}=h_{\mathrm{so}}+Cp_{s}(T-T_{0})$$

(10) $$s_{s}=s_{\mathrm{so}}+Cp_{s}\ln\left(\frac{T}{T_{0}}\right)$$

(11) $$h_{w}=h_{\mathrm{wo}}+Cp_{w}(T-T_{0})$$

(12) $$s_{w}=s_{\mathrm{wo}}+Cp_{w}\ln\left(\frac{T}{T_{0}}\right)$$

It is worth mentioning that the enthalpy and entropy are dependent on temperature but independent of pressure [16]. The specific heat of salt (*Cp*_s_) at 25 °C is 0.8368 (kJ/kg K), while the specific heat of water (*Cp*_w_) at 25 °C (dead state) is 4.1816 (kJ/kg K). Moreover, the enthalpy and entropy of salt and water at 25 °C are *h*_so_ = 21.0455 (kJ/kg) and *s*_so_ = 0.07328 (kJ/kg K), *h*_wo_ = 104.86 (kJ/kg), and *s*_wo_ = 4.180 (kJ/kg K), respectively [1].

The entropy of saline water per unit mass in an ideal solution at any temperature *T* (K) and pressure P (kPa) is specified by:

(13) $$s=mf_{s}\cdot s_{s}(T,P)+mf_{w}\cdot s_{w}(T,P)-R\cdot (x_{s}\ln(x_{s})-x_{w}\ln(x_{w}))$$

R is the gas constant, at 8.314 (kJ/kmol K).

The exergy *Ex*_gy_ (kJ/kg) of a flow stream is given as [1,27]:

(14) $$Ex_{\mathrm{gy}}=(h-h_{0})-T_{0}(s+s_{0})$$

Additionally, the rate of exergy flow rate related to a fluid stream is given by Equation (15):

(15) $$X^{o}=m^{o}\cdot Ex_{\mathrm{gy}}=m^{o}\left[\left(h-h_{0})-T_{0}(s+s_{0}\right)\right]$$

The model equations are coded within the gPROMS software suite and then solved to evaluate the specific exergy and exergy flow rates at different locations throughout the RO system. Afterwards, the exergy flow rates of the specified locations will be used to evaluate the exergy destructed within any selected unit, component, and stream via the evaluation of an exergy balance.

### 2.3. Discussion of Exergy Distribution of the RO System

The specific exergy, exergy rate and the rate of exergy change at all major states for each component of RO desalination plant are listed in Table 3. The selected locations of the states for each component are numbered in the schematic diagram of the RO desalination plant in Figure 1.

The exergy at position (0) is zero since there is no energy consumption at this point. However, at position (1), the high-pressure pump provides the system with energy to work at the dead state, (25 °C, 9.22 atm, 950.16 ppm, and 17.72 kg/s). Point (1) presents the feed brackish raw water stream of the plant before being connected with the retentate plant stream at point (2), where the exergy rate is 25.163 kJ/kg. This pump is not continually used in the RO system of the APC since it is only used to draw water from a well and for pumping it into the RO system. The RO system contains four high pressure pumps, two in the first pass and two in the second pass. It should be noted that the exergies of retentate (brine) streams are negative because of higher salinity than the dead state level.

The brackish water stream enters the RO system at a temperature of 30 °C and a pressure of 9.22 atm, and the output streams are the permeate and retentate that exit at the same temperature but with different salinities. As shown in Table 2, there is a total of 9.432 kW exergy input to the system through the pumps. About 67.8% of the exergy is destroyed by the group A of the first pass RO process at position (3,4), and the residual 32.2% is contributed by the high-pressure pumps of group A of the second pass RO process at position (19,20).

The exergy of raw brackish water at position (2) which presents a mixing point will be assigned as zero since it indicates the dead state [16]. However, Figure 1 shows that the feed raw water relates to a recycled retentate stream of 2.77 kg/s. At the connection point (position 2), the measured temperature confirmed an increase from 25 °C to 30 °C which causes an increase in the exergy rate by 16.699 kJ/kg. This is basically associated with an increase in the raw water salinity to 1098.62 ppm and the total feed plant flow rate to 20.49 kg/s. A total 9.31% of the input exergy is destroyed (Table 3) due to the mixing of the recycled stream of the second pass and the feed raw water at position (2). This is expected since a mixing dot (position 2) can yield work when solutions of different concentrations are mixed reversibly. Position (2) represents a reversible mixing point where low salinity water is mixed with a high salinity water and therefore reversible work could be supplied. Therefore, the destructed exergy in the mixing process characterizes the produced work for a reversible mixing process [28].

Positions (5–16) represent the first pass RO membrane components where the feed brackish water separates to the permeates at (5, 7, 9, 11, 13, and 15) and the retentate at positions (6, 8, 10, 12, 14, and 16), whereupon 28.26% of the total exergy input is destroyed by RO permeate streams and 41.33% by RO retentate streams. Table 3 shows that the pressure decreases from 9.22 atm to 8.94 atm in the separation process (the first pass). The decreases in brine pressure causes an increase in the dissipated exergy, which means the exergy destroyed by retentate streams is greater than that destroyed by permeate.

Regarding the second pass of RO system, the permeate product from the first pass is pumped by high pressure pumps to the second pass at positions 21 to 28. This point indicates the total input exergy. The fed permeate water is processed in the second stage and produces the high-quality water at positions 21, 23, 25, and 27 and retentates at positions 22, 24, 26, and 28. This would interpret the dissipated exergy of the total exergy input of 69.39% caused by the permeate streams and 71.36% by retentate streams. This can be attributed to a decrease in the brine pressure, which results in greater exergy loss in retentate streams than in the permeate ones. The outgoing retentate at position (27′) leaves the system at the dead state of system (25 °C and 9.22 atm) with a salinity of 409.2 ppm and has a negative exergy rate of −0.087 kW. The negative sign is evidence that the work input to the brine is essential to drive the brine into its dead state. Note, this point considers as a mixing point of retentate collected from stages A and B. However, the product permeate water at position 30 has a positive exergy rate of 6.04 kW with the capacity of generating work relative to the dead state. In this regard, the net exergy discharge represents the variance between the exergies of the retentate and the product water, which is equal to 2.91 kW. Specifically, this amount is the lowest work requirement to extract product water at a mass flow rate of 13.85 kg/s and 2 ppm salinity from the feed saline water of 1098.62 ppm flowing at a mass flow rate of 20.49 kg/s.

The net exergy discharge is another feasible tool to represent the net salinity exergy discharge because of its relation to the salinity variation. Table 3 shows positive and negative values of the rate of exergy change of components. This is also a clear indication of transferred exergy to component (positive) and destroyed exergy by component (negative).

It can be seen from Table 3 that the largest exergy losses arise in the RO process desalination plant at position (17) of the mixed permeate streams and at position (30) of the mixed product streams with rates of 5.88 kW and 9.03 kW, respectively. This accounts for 62.28% and 95.74% of the total exergy input, respectively. Furthermore, the disposed retentate stream at position (18) and the mixing point of retentate streams of plant at position (27′) have rates of 7.57 kW, and 6.13 kW, respectively, which accounts for 71.18%, and 64.95% of the total exergy input, respectively. These results are quite different than the ones presented by El-Emam and Dincer [1] and Cerci [16], that indicated that the membrane modules are responsible for exergy losses of around 67.8% and 74%, respectively, from the total exergy input. This can be attributed to different scale size and design of the RO system besides using seawater. It can be stated that seawater desalination consumes more specific energy compared to brackish water desalination [18].

The negative exergy rate of −4.33 kW of the disposal retentate at position (18) can be attributed to the work input to the retentate to convert it from 29.9 °C and 8.68 atm to the dead state at 25 °C and 9.22 atm, besides the high salinity of discharged retentate water of 4426.27 ppm.

Table 3 also shows that the brine pressure in the throttling valve at positions (31, 32) decreases from 13.8 atm to the dead state pressure of 9.22 atm, resulting in 2.99 kW of exergy destruction, which amounts to 31.71% of the exergy input.

The second law of efficiency of the RO desalination plant is estimated via the division of the net salinity exergy by the total exergy input supplied by the first and second passes pumps, i.e.,

(16) $$Efficiency=\frac{Net\_Exergy\_rate}{Total\_Exergy\_Input}\cdot 100$$

From the above relation, the efficiency of the first pass of RO system is 1.86%, whilst that of the second pass is 2.63%. This indicates that the RO system at the given throughput can produce water of acceptable purity using only 4.48 kW of exergy instead of 9.43 kW.

In a fact, most of the exergy input to the RO system is depleted in the components, while the residual exergy is discharged from the system. The pressure drops in the mixing points, the membrane modules, the brine transmission streams, and the throttling valve are the main causes of the exergy destruction. Based on this, it can be said that the different results of exergies for outgoing streams depend on the degree of salinities.

**Figure 2:**
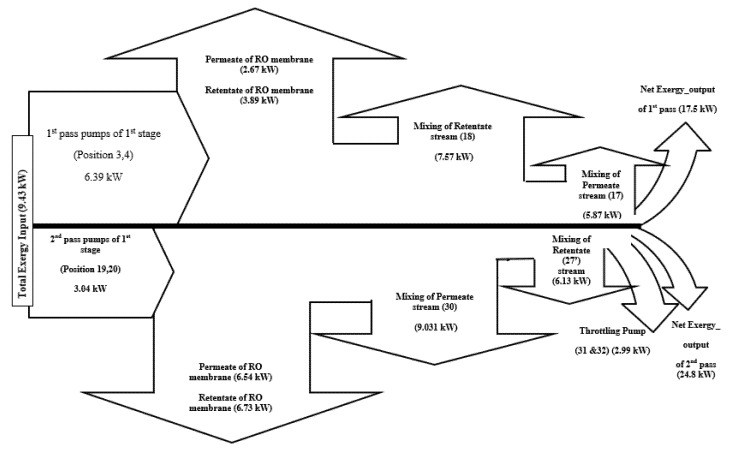
Rate of exergy change flow chart of the RO system of the APC. Note: Positive values of rate of exergy change specify input exergy, while negative values specify destroyed rate of exergy change.

Figure 2 shows the rate of exergy change flow chart of the RO system and the concluded results of input and destructed exergy. This is specifically referring to the rate of exergy change (ΔX, kW) for the whole plant. The product stream of the plant (Position 30) and the disposed retentate stream of the first pass (Position 18) are responsible for causing the highest exergy destructions in the RO system with exergy rates of 9.03 kW and 7.57 kW, respectively. Statistically, these findings account for 95.8% and 71.18% of the total exergy input. Additionally, the recycled retentate stream of the desalination plant (Position 27′) and the mixing location of permeate of the first pass (Position 17) cause exergy rate destructions of 6.13 kW and 5.87 kW, respectively. These precisely account for 64.95% and 62.28%, respectively, of the total exergy input.

Exergy distribution throughout the plant components is derived from Table 3, and is shown in Figure 2. As seen from the figure, there is a total of 9.43 kW exergy input to the system through the pumps, with 6.39 kW, which is about 67.8% of the exergy input, supplied by the high pressure pump of (first pass pumps of the first stage, group A) at positions 3,4, and the remaining 3.04 kW, which is about 32.2% of the exergy input, supplied by the high pressure pump of (second pass pumps of the first stage, group A) at positions 19,20. The related results of exergy destruction in the second pass pumps are in accordance with the findings of Kahraman et al. [23]. Kahraman et al. [23] analysed the exergy destruction of a brackish water desalination system and stated that 39.7% of the total exergy is destructed in pumps.

The net output exergy for the first pass, which is calculated from the summation of exergy rate X (kj/kg) of the first pass (location 2 to 18), is about 17.5 kW (Figure 2), which assures an efficiency equivalent to 1.86% from the total input 9.43 kW. This is already calculated from Eq. 16. In addition, the net output exergy for the second pass, which is calculated from the summation of exergy rate X (kj/kg) of the second pass (location 19 to 30), is 24.8 kW, with an efficiency equivalent to 2.63% from the total net output exergy of the whole input exergy of 4.48%.

To quantify the issue of thermal losses of the brackish water RO desalination system of the APC, several recommendations are made:

- Using highly water permeable membranes of higher membrane surface area would improve the productivity;
- Optimise the RO process to investigate the optimal operating condition that suits the lowest pressure drop throughout the modules;
- Utilise an energy recovery device of a high efficiency to absorb the surplus energy from the brine stream;
- Replacing the existing pumps with higher efficiency pumps;
- Scale-up the RO process into a larger size. Basically, a large scale RO process consumes lower energy per kg of pure water produced, compared to a small size RO system;
- Maintain a scheduled membrane cleaning regime to prevent the propensity of fouling.

This study has investigated the locations of high exergy destruction in a desalination plant, which can be efficiently exploited to advance the thermodynamic performance of the plant. The obtained results of this study are in a full agreement with the findings of other studies carried out on different RO desalination systems. In this regard, the RO system has a second law efficiency of 4.48%, which is close to the second law efficiency presented in [16]. However, it is important to realize that all the exergy calculations are carried out based on the hypothesis of ideal solutions of water and salt (brackish water). In addition, it is important to mention that the RO system of the APC has used new membranes at the time of collecting experimental measurements of the RO plant. This introduced no fouling propensity. In this regard, the model developed by the same authors [11] has considered the fouling parameter as 1 due to the use of new membranes in the RO modules. Thus, the fouling influence on the thermodynamic limitations and exergy analysis has not been considered in this study.

## 3. Conclusions

This paper focused on the calculation of exergy analysis based on thermodynamic limitations of the multistage multi pass brackish water RO desalination plant of the APC with a daily production rate of 13.85 kg/s. The exergy calculations have considered both physical and chemical exergies of the RO desalination plant. To carry out this aim, a comprehensive set of thermodynamic equations was embedded in the model of the RO system (developed by the same authors) to carry out the analysis of exergy destruction. In this regard, a computational code was developed using gPROMS software to analyze the system and asses its performance.

Several locations throughout the RO system were selected and subjected to the calculations of exergy destruction to identify the locations of largest energy losses. The results of this study indicated that the product stream and the disposed retentate stream of the first pass are responsible for causing the highest exergy destructions in the RO system with exergy rates of 9.03 kW and 7.57 kW, respectively. Statistically, these findings account for 95.8% and 71.18% of the total exergy input. In addition, the recycled retentate stream of the desalination plant and the mixing location of permeate of the first pass cause exergy rate destructions of 6.13 kW and 5.88 kW, respectively. These precisely account for 64.95% and 62.28%, respectively, of the total exergy input.

## Figures and Tables

**Table 1:** Characteristics of the spiral wound membrane element and transport parameters [11].

Parameter	Value
Membrane type and configuration	TMG20D-400, ultra low pressure BWRO, spiral wound, polyamide thin-film composite
Feed and permeate spacer thickness	8.6 × 10^−4^ (34 mils), 5.5 × 10^−4^ (m)
Hydraulic diameter of the feed spacer channel	8.126 × 10^−4^ (m)
Membrane area	37.2 (m^2^)
Maximum operating pressure	40.464 (atm)
Maximum operating temperature	45 (°C)
Minimum salt rejection	99.5%
Water transport parameter at 25 °C	9.6203 × 10^−7^ (m/atm s)
Solute transport parameter at 25 °C	1.61277 × 10^−7^ (m/s)
Spacer type	NALTEX-129
length of filament in the spacer mesh	2.77 × 10^−3^ (m)

**Table 2:** The dead state operating conditions of the RO desalination plant of the APC.

Temperature (°C)	Pressure (atm)	Salinity (ppm)	Flow Rate (kg/s)
25	9.22	1098.62	20.49

**Table 3:** Rate of exergy change of major components of the RO system of the APC desalination plant.

Component	Location	Temperature (°C)	Pressure (kPa)	Mass Rate (kg/s)	Salinity (ppm)	Chemical Exergy (kJ/kg)	Physical Exergy (kJ/kg)	Specific Exergy Ex (kJ/kg)	Exergy Rate X (kJ/kg)	Rate of Exergy Change ΔX (kW)	ΔX (kW)
Pump for tank	0	25	101.32	17.72	950.16	0	0	0	0	25.162	25.162
Pump for tank	1	25	934.22	17.72	950.16	−0.08	1.5	1.42	25.1624	−17.577	--
Mixing with opening valve	2	30	934.22	20.49	1098.62	−0.1037	0.4739	0.3702	7.5853	−0.8777	0.8778
Before 1st pass pump 1st stage	3	25	923.07	10.25	549.31	−0.0531	0.7075	0.6544	6.7076	0.0030	0.0031
After 1st pass pump 1st stage	4	30	934.22	10.25	549.31	−0.0529	0.7076	0.6547	6.7106	−6.3939	6.3939
Permeate of RO membrane	5	29.6	934.22	0.213	60.49	−0.0062	1.4931	1.4869	0.3167	−0.5500	0.5500
Retentate of RO membrane	6	29.6	905.85	1.142	2414.17	−1.495	1.2907	−0.2043	−0.2333	0.3826	0.3826
Permeate of RO membrane	7	25	934.22	0.213	60.49	−0.0061	0.7071	0.701	0.1493	−0.3833	0.3833
Retentate of RO membrane	8	30	905.85	1.142	2414.17	−1.5767	1.3718	−0.2049	−0.2339	0.3833	0.3833
Permeate of RO membrane	9	25	934.22	0.213	60.49	−0.0061	0.7071	0.701	0.1493	−0.3831	0.3832
Retentate of RO membrane	10	29.9	905.85	1.142	2414.17	−1.5561	1.3513	−0.2048	−0.2338	0.3831	0.3832
Permeate of RO membrane	11	25	934.22	0.213	60.49	−0.0061	0.7071	0.701	0.1493	−0.3831	0.3832
Retentate of RO membrane	12	29.9	905.85	1.142	2414.17	−1.5561	1.3513	−0.2048	−0.2338	0.3354	0.3354
Permeate of RO membrane	13	25	934.22	0.147	163.55	−0.0163	0.7071	0.6908	0.1015	−0.4829	0.4829
Retentate of RO membrane	14	29.9	879.50	1.221	4426.27	−1.5575	1.2451	−0.3124	−0.3814	0.4829	0.4829
Permeate of RO membrane	15	25	934.22	0.147	163.55	−0.0163	0.7071	0.6908	0.1015	−0.4829	0.4829
Retentate of RO membrane	16	29.9	879.5	1.221	4426.27	−1.5575	1.2451	−0.3124	−0.3814	1.9309	1.9309
Mixing of permeate stream	17	30	934.22	4.258	106.89	−0.0109	0.3748	0.3639	1.5494	−5.8744	5.8745
Mixing of retentate stream and disposal	18	25	934.22	4.882	4426.27	−1.006	0.1201	−0.8859	−4.3249	7.5659	7.5659
Before 2nd pass pump 1st stage	19	30	879.50	2.129	534.5	−0.053	1.575	1.522	3.241	0	0
After 2nd pass pump 1st stage	20	30	996.03	2.129	534.4	−0.053	1.575	1.522	3.241	−3.035	3.035
Permeate of RO membrane	21	25	934.22	0.291	2.00	−0.0002	0.707	0.707	0.206	1.875	1.875
Retentate of RO membrane	22	30	933.2	2.104	106.2	−1.575	2.564	0.989	2.081	−1.488	1.488
Permeate of RO membrane	23	25	934.22	0.291	2.00	−0.0002	2.037	2.037	0.593	2.059	2.059
Retentate of RO membrane	24	30	933.20	2.104	106.2	−1.303	2.564	1.260	2.6526	0.568	0.567
Retentate of RO membrane	25	30	856.19	2.568	172.2	−1.303	2.557	1.254	3.219	−2.686	2.686
Permeate of RO membrane	26	25	934.22	0.262	3.66	−0.0004	2.037	2.037	0.534	1.989	1.989
Retentate of RO membrane	27	30	856.19	2.568	172.2	−1.575	2.557	0.982	2.523	−1.988	1.988
Permeate of RO membrane	28	25	923.07	0.262	3.66	−0.0004	2.04	2.039	0.534	−0.621	0.621
Mixing of retentate stream	27′	30	934.22	2.118	409.2	−1.554	1.514	−0.041	−0.087	6.127	6.126
Mixing of permeate stream or product	30	30	934.22	13.85	2.00	0.011	0.425	0.436	6.039	−9.031	9.031
Throttling valve	31	30	1400	2.118	409.2	−1.453	0.041	−1.412	−2.991	2.991	2.991
Throttling valve	32	30	1400	2.118	409.2	0	0	0	0	0	--

## Data Availability

Not applicable.
